# Errors in Citations and Tables 1 and 4

**DOI:** 10.1001/jamahealthforum.2026.2551

**Published:** 2026-07-10

In the Original Investigation titled “Regulatory Definitions and Classification of Biosimilar Medications Across the 6 Regions of the World Health Organization: A Scoping Review”^1^ published online on May 31, 2026, in *JAMA Health Forum*, a number of citations to sources were incorrectly numbered; information for Tanzania was inaccurate in Table 1; and fewest number of abbreviated pathway items in Table 4 should have been 0. This article was corrected online.
